# Supplementary material for: Sensitivity and Specificity of two rapid tests for the diagnosis of infection by Trypanosoma cruzi in a Colombian population
Source: PLoS Negl Trop Dis. 2021 Jun 2;15(6):e0009483. doi: 10.1371/journal.pntd.0009483 (PMC8202949; doi:10.1371/journal.pntd.0009483)
Supplement: Attachment — Submitted filename: Letter_REVFHH2.docx [file pntd.0009483.s004.docx]

March 31^th^, 2021

To: Editors

PLOS Neglected Tropical Diseases

RE: Article Review PNTD-D-20-02109: “Evaluation of two rapid tests for the diagnosis of Trypanosoma	cruzi infection in a Colombian population”.	“Evaluation of two rapid tests for Trypanosoma cruzi”.

First, the authors would like to thank the reviewers and the editors for their clear, concise comments. It is important to note that our corrections were completed following the recommendations of the reviewers.

We hope that we have adequately addressed the issues they raised. We are thankful for their time and their attention to detail. Below you will find our responses to their evaluations.

Sincerely,

Sandra Suescún-Carrero

Corresponding author

We want to note in addition to reviewers' comments that the paper should be modified according to the STARD guidelines for the reporting of diagnostic studies (see <https://www.equator-network.org/wp-content/uploads/2015/03/STARD-2015-checklist.pdf>), beginning from the title and abstract. Be more concise, but linked to guidelines.

**Title**

*Answer*: The title was modified in accordance with the STARD guidelines.

Long title: The word “Evaluation” was removed and replaced by the phrase “Sensitivity and Specificity” (L 4 a 5).

Evaluation of two rapid tests for the diagnosis of *Trypanosoma cruzi* infection in a Colombian population

Sensitivity and Specificity of two rapid tests for the diagnosis of infection by *Trypanosoma cruzi* in a Colombian population

Short title: The Word “Evaluation” was removed and replaced by “Sensitivity and Specificity” (L 8).

Evaluation of two rapid tests for *Trypanosoma cruzi*

Sensitivity and Specificity of two rapid tests for *Trypanosoma cruzi*

**Abstract**

*Answer*: The Abstract was modified according to the STARD guidelines.

Modified Abstract: (L 12 a 35).

**Objective.** To evaluate diagnostic precision of two rapid diagnostic tests (RDT's) on patients with chronic Chagas disease.

**Methodology.** Prospective study with the following inclusion criteria: subjects older than 3 years, signed informed consent. Exclusion criterion: subjects could not have previously received treatment for infection with *T. cruzi*. The study population were participants in a screening process undertaken in rural and urban zones of the department Boyacá, Colombia. Two RDT’s were performed to all participants: the Chagas Detect Plus InBios (CDP) and the Chagas Stat-Pak (CSP) and as a reference standard the ELISA Chagas III GrupoBios and the Chagas ELISA IgG+IgM I Vircell tests were used. In the case of discordant results between the two ELISA tests, an indirect immunofluorescence was done.

**Results.** Three hundred-five (305) subjects were included in the study (38 patients with leishmaniasis), of which 215 tested negative for *T cruzi* and 90 tested positive according to the reference standard. The sensitivity of the RDT’s were 100% (CI 95% 95.9 – 100), and the specificity of the CDP was 99.1% (CI 95% 96.6 – 99.8) and for CSP was 100% (CI 95% 98.3 – 100). The agreement of CDP was 99.5% and for CSP was 100% with Kappa values of (k=99.1; CI 95% 92.6-99.8%) and (k=100; CI 95% 94.3-100), respectively. RDT’s did not present cross-reactions with samples from patients who were positive for leishmaniasis.

**Conclusions**

The findings demonstrate excellent results from the RDT’s in terms of validity, safety, and reproducibility. The results obtained provide evidence for the recommendation for using these tests in a Colombian epidemiological context principally in endemic areas in which laboratory installations necessary to perform conventional tests are not available, or they are scarce and to help in diagnosing chronic Chagas disease in order to provide access to treatment as soon as possible.

Previous Abstract:

**Background**

Chagas is caused by the parasite *Trypanosoma cruzi,* and is one of the most important public health concerns affecting Latin America. The acute infection is asymptomatic, adiagnosed at a rate of only 1 to 2%. Most cases are diagnosed during the chronic phase in which the diagnostic techniques of choice are serological and use two enzyme-linked immunosorbent assay (ELISA) tests, which have high degrees of sensitivity and specificity; however, these types of tests are not practical in many endemic regions because many local laboratories are ill-equipped to carry them out. Rapid Diagnostic Tests (RDT) also exist for the detection of *Trypanosoma cruzi* infections which are easy to use; only require small sample quantities; and do not require specialized laboratory personnel, electrical equipment, or refrigeration. This study evaluated the validity and safety of two RDT's, using ELISA tests as a reference.

**Methodology/Results**

The RDT's were performed in both rural and urban zones in the department of Boyacá, Colombia, while ELISA reference tests were completed at the Boyacá Departmental Laboratory of Public Health. Three-hundred five subjects were studied, of which 38 patients presented leishmaniasis. The Chagas Detect Plus (CDP) (InBios International Inc., Seattle, USA) and the Chagas Stat-Pak (CSP) (Chembio Inc., Medford USA) RDT’s were used. Both had sensitivities of 100%, while their specificities were 99.1% and 100%, respectively. Their concordances were 99.5% (CDP) and 100% (CSP), which agreed with their respective Kappa values (k=99.1; IC 95% 92.6-99.8%) and (k=100; IC 95% 94.3-100). There were no cross reactions between samples in patients who were positive for leishmaniasis.

**Conclusions**

The results obtained support the use of RDT's in the Colombian epidemiological context to help diagnose chronic Chagas disease in order to improve access to treatment as soon as possible for the populations studied.

Methodology seems solid but reporting should be improved and the issue of the leishmania positive samples should be explained well (It seems that you added leishmania positive semples to the analysis but in fact you found some individuals with positive serology (?) to leishmania in the study population).

*Answer*: More clarity was provided regarding the methodology used and in terms of matters related to patients with leishmaniasis.

In order to evaluate cross-reactivity with leishmaniasis, participants from the municipalities of Otanche and San Pablo de Borbour were convened, they were diagnosed by the program of diseases transmitted by vectors of the LDSP with cutaneous leishmaniasis confirmed through the identification of the parasites from smears from cutaneous lesions using microscopy (L 190 a 194 ).

Answers to questions posed by reviewers.

----------------------------------------------------------------------------------------

**Methods**

**Reviewer #1:** The sample size was sufficient to ensure the quality of the results and the statistical analysis were correct.

The work was well executed and contain important information about the use of rapid diagnostic tests (RDT) to facilitate the diagnosis of Chagas Disease (CD) in regions where the laboratory infrastructure is poor.

The authors declare that among the samples used, some were positive for leishmania, but I did not find the serological criteria or methodology used to define positivity for leishmania. It would be important to make it very clear how they concluded with leishmania positivity.

*Answer*: We accept the reviewer’s recommendation. The following paragraph was included:

In order to evaluate cross-reactivity with leishmaniasis, participants from the municipalities of Otanche and San Pablo de Borbour were convened, they were diagnosed by the program of diseases transmitted by vectors of the LDSP with cutaneous leishmaniasis confirmed through the identification of the parasites from smears from cutaneous lesions using microscopy (L 190 a 194 ).

**Reviewer #2:** The study was performed in seven municipalities with high endemicity for CD and included 2 areas endemic for leishmaniasis. Study design appears to be adequate and compared the accuracy of 2 RDTs with the ELISA and IFI techniques performed in laboratories that are certified by the Bolivian MoH. Study development was according to the approved protocol, including reaching the programed sample size.

1) Sample size determination needs in my view a clarification on the meaning of the sentence: “a sample of 305 people was required that included individuals without infections by T. cruzi, but with leishmaniasis”.

*Answer*: We accept the reviewer’s recommendation. The phrase “a sample of 305 people was required that included individuals without infections by *T. cruzi*, but with leishmaniasis” was replaced with the phrase “the necessary sample size was of 305 subjects”, as it caused confusion, and as in the previous paragraph it was explained that endemic Leishmaniasis zones were included (L 151 a 153).

2) Also it is not clear if Otanche and San Pablo Borbur are co-endemic for CD and Leish.

*Answer*: We accept the reviewer’s recommendation. We clarified that the municipalities of Otanche and San Pablo de Borbur are only endemic for Leishmaniasis and not for infection by *T. cruzi* (L 147).

3) The clinical form of leishmaniasis should be indicated as well as what diagnostic test was used.

This was the same observation made by Reviewer #1.

*Answer*: We accept the reviewer’s recommendation.

In order to evaluate cross-reactivity with leishmaniasis, participants from the municipalities of Otanche and San Pablo de Borbour were convened, they were diagnosed by the program of diseases transmitted by vectors of the LDSP with cutaneous leishmaniasis confirmed through the identification of the parasites from smears from cutaneous lesions using microscopy (L 190 a 194 ).

4) Please also clarify the sentence:  “in the present study, the ELISA Chagas III GrupoBios® test was used, and as a complementary test, according to the recommendations of INS, the recombinant antigen or synthetic peptide ELISA test was used with a specificity of ≥ 98%, after first using a Chagas ELISA IgG+IgM I Vircell® test”. Which test is applied first?

*Answer*: We accept the recommendation of the reviewer for more clarity.

The paragraph below was replaced:

which recommends that the first test (total/raw antigen ELISA) used in the sample algorithm confirms a sensitivity ≥ 98%; because of this, in the present study, the ELISA Chagas III GrupoBios® test was used [29], and as a complementary test, according to the recommendations of INS, the recombinant antigen or synthetic peptide ELISA test was used with a specificity of ≥ 98%, after first using a Chagas ELISA IgG+IgM I Vircell® test [30,31]. In the case of conflicting results, as a third proof of principal or of different antigens than the first two tests, an IgG antibody technique for *T. cruzi* was used for IFI [32]: when the results of this technique were positive, the case was considered to be confirmed. All tests were completed using the serum following the manufacturer's instructions and the results were interpreted as either positive or negative.

By the paragraph:

All samples were analyzed using the ELISA Chagas III GrupoBios® tests [informed sensitivity, 100%; specificity, 100%] [29] and with the Chagas ELISA IgG+IgM I Vircell® tests [informed sensitivity, 100%; specificity, 98%] [30,31]. In cases in which discordant results were found between the two ELISA tests, an IIF test was done [32], which uses epimastigotes of the Colombian *T. cruzi* strains DTU and TcI as antigens, when the results of this technique were positive, case was considered to be confirmed. The tests were done in serum samples in the LDSP, following the manufacturer’s instructions (L 172 a 179 ).

**Reviewer #3:**

1) The methodology for establishing positive infection (lines 165-177) are problematic and difficult to follow (although they are similar to what is unfortunately used with some frequency).  % senstitivity is given for the GrupoBios test – how was this number determined (using what sets of samples?).  What are the sensitivities of the Vircell and the “recombinant antigens or synthetic peptide” tests (perhaps this refers to the same thing?)?  Why is one of these referred to as “complementary”?  Although hard to understand, it sounds like 3 tests were done and if there were “conflicting results” (what does conflicting results mean – all not positive or negative?) then an IFA was done.  Why is this referred to as a “proof of principal” test?

This was the same as the observation of Reviewer #2.

*Answer*: We accept the recommendation of the reviewer for better clarity.

The following paragraph was replaced:

which recommends that the first test (total/raw antigen ELISA) used in the sample algorithm confirms a sensitivity ≥ 98%; because of this, in the present study, the ELISA Chagas III GrupoBios® test was used [29], and as a complementary test, according to the recommendations of INS, the recombinant antigen or synthetic peptide ELISA test was used with a specificity of ≥ 98%, after first using a Chagas ELISA IgG+IgM I Vircell® test [30,31]. In the case of conflicting results, as a third proof of principal or of different antigens than the first two tests, an IgG antibody technique for *T. cruzi* was used for IFI [32]: when the results of this technique were positive, the case was considered to be confirmed. All tests were completed using the serum following the manufacturer's instructions and the results were interpreted as either positive or negative.

By the paragraph:

All samples were analyzed using the ELISA Chagas III GrupoBios® tests [informed sensitivity, 100%; specificity, 100%] [29] and with the Chagas ELISA IgG+IgM I Vircell® tests [informed sensitivity, 100%; specificity, 98%] [30,31]. In cases in which discordant results were found between the two ELISA tests, an IIF test was done [32], which uses epimastigotes of the Colombian *T. cruzi* strains DTU and TcI as antigens, when the results of this technique were positive, case was considered to be confirmed. The tests were done in serum samples in the LDSP, following the manufacturer’s instructions (L 172 a 179 ).

2) ¿What were the antigens used in the IFA?

*Answer*: We accept the reviewer’s recommendation. The following was included:

In cases in which discordant results were found between the two ELISA tests, an IIF test was done [32], which uses epimastigotes of the Colombian *T. cruzi* strains DTU and TcI as antigens, when the results of this technique were positive, case was considered to be confirmed (L 175 a 178 ).

3) Lines 216-219 refer to negative and positive and indicate that no “indeterminate results” were observed.  What constitutes “conflicting samples”?  It should be more clear how +/- was determined and what would be defined as “indeterminate”.

*Answer*: We accept the recommendation of the reviewer. The following phrase was omitted because it is confusing: “None of the participants presented indeterminate results” (L 225).

The word “conflicting” was replaced with the word “discordant”.

Two (2) discordant samples were obtained by the RDT's, and these were found to be negative by the reference tests (Figure 1) (L 226).

4) Ideally, a supplemental table of all the test results for each sample, including quantitative results, should be supplied.

*Answer*: We accept the reviewer’s recommendation. A supplementary table of all the qualitative and quantitative results of the rapid diagnostic tests and the tests used as reference standards was provided.

**S1** Complimentary table of qualitative and quantitative results of rapid diagnostic tests and standard reference tests utilized in the study (L 627 a 628).

5) How was Leishmania infection determined?

This was the same as the observation of Reviewer #1.

*Answer*: We accept the recommendation of the reviewer.

In order to evaluate cross-reactivity with leishmaniasis, participants from the municipalities of Otanche and San Pablo de Borbour were convened, they were diagnosed by the program of diseases transmitted by vectors of the LDSP with cutaneous leishmaniasis confirmed through the identification of the parasites from smears from cutaneous lesions using microscopy (L 190 a 194 ).

6) Although difficult to understand the pre-screening protocol, it sounds like all sera got tested initially with 2 or 3 tests (this is not clear) and if positive on one of these, then an IFA was done and if the IFA was positive, then the serum was considered positive – and if negative, was excluded from testing with the RDA.  It seems, as in many evaluations, “positive” sera are marked as positive if they are positive on multiple other tests while those that test positive on only 1 test, are excluded.  However, this does not resolve the discordant sera – and means that sera are being selected for DRT testing ONLY if they are strongly positive on multiple tests.  Borderlines – even if truly positive are not further evaluated.  This skews the results for the RDT.  This is a common way of doing these types of evaluations but it gives overly optimistic data on the tests being evaluated – 100% sensitivity in this case.  Which is quite unlikely given that the tests used to initially

grade the sera are only 98% sensitive or less.  At a minimum, the authors should note that the evaluated tests have 100 sensitivity using a highly selected set of sera.

*Answer*: We accept the recommendation of the reviewer for more clarity related to the methodology.

This is the same observation made by Reviewer #2.

The following paragraph was replaced:

which recommends that the first test (total/raw antigen ELISA) used in the sample algorithm confirms a sensitivity ≥ 98%; because of this, in the present study, the ELISA Chagas III GrupoBios® test was used [29], and as a complementary test, according to the recommendations of INS, the recombinant antigen or synthetic peptide ELISA test was used with a specificity of ≥ 98%, after first using a Chagas ELISA IgG+IgM I Vircell® test [30,31]. In the case of conflicting results, as a third proof of principal or of different antigens than the first two tests, an IgG antibody technique for *T. cruzi* was used for IFI [32]: when the results of this technique were positive, the case was considered to be confirmed. All tests were completed using the serum following the manufacturer's instructions and the results were interpreted as either positive or negative.

By the paragraph:

All samples were analyzed using the ELISA Chagas III GrupoBios® tests [informed sensitivity, 100%; specificity, 100%] [29] and with the Chagas ELISA IgG+IgM I Vircell® tests [informed sensitivity, 100%; specificity, 98%] [30,31]. In cases in which discordant results were found between the two ELISA tests, an IIF test was done [32], which uses epimastigotes of the Colombian *T. cruzi* strains DTU and TcI as antigens, when the results of this technique were positive, case was considered to be confirmed. The tests were done in serum samples in the LDSP, following the manufacturer’s instructions (L 172 a 179 ).

In the following paragraph, this phrase was added: Thus, in the present study, all samples were analyzed using…

Thus, in the present study, all samples were analyzed using two immunochromatographic diagnostic tests based on different antigens: Chagas Detect Plus (InBios International Inc., Seattle, USA) ® CDP [35], which uses a multi-epitope antigen, and Chagas Stat-Pak (Chembio Inc., Medford USA) ® CSP [36], which employs a combination of recombinant proteins. Small quantities of whole blood from finger puncture were used to carry out the RDT's (L 185 a 190 ).

Figure 1 was also fixed: Flowchart of study participants and RDT and ELISA test results. To provide more clarity in showing that the two participants with discordant results in the reference tests were not excluded from the study. The two reference tests and the two RDT’s were done for all 305 participants in the study. In the two cases that returned discordant results in the reference tests, the IIF test was administered according to the diagnostic algorithm for chronic CD in Colombia.

--------------------------------------------------------------------------

**Results**

**Reviewer #1:** Results were clearly and completely presented, but I think that Legends of tables #1 and #2 can be improved to facilitate the interpretation.

*Answer*: We accept the recommendation of the reviewer. We changed the Legends of tables #1 and #2 as follows:

**Table 1.** Characterization of the population included in the study (n= 305)

**Table 2.** Discordant results obtained from EC tests in patients from the department of Boyacá

**Reviewer #2:** Results are clearly presented. They show that the 2 RDTs performed well in the study sites, with high specificity, sensitivity, PPV, and NPV. When compared to the “gold standard” techniques, the RDTs showed high concordance and fewer discrepancies. Cross reactions in patients with leishmaniasis were not observed.

The results of the present study favorably compare to other existing studies of RDTs’ performance in different endemic and non-endemic regions.

*Answer*: Thank you for the comment.

**Reviewer #3:** (No Response)

---------------------------------------------------------------------------------

**Conclusions**

**Reviewer #1:** It is important to reinforce the quality control conditions in which the work was carried out.

*Answer*: We accept the recommendation of the reviewer. The internal and external quality controls utilized in the laboratory were described as follows:

The tests were done in serum samples in the LDSP, following the manufacturer’s instructions; the laboratory complies with the internal quality control standards of the method, using material for quality control of the MQC and with external quality controls and aptitude tests done by the INS and Proasecal. The results of the tests were interpreted as either positive or negative. (L 178 to L 182).

**Reviewer #2:** Conclusions are supported by the data. The limitations of the study are indicated, including the low number of patients with leishmaniasis.

The data obtained contributes to the possible implementation of better performing diagnostic tests and algorithms for CD.

The potential and benefit of introducing the studied RDTs in the Colombian setting are discussed, that could result in better patient management and reduced morbidity and mortality.

*Answer*: Thank you for the comment.

**Reviewer #3:** (No Response)

---------------------------------------------------------------------------

**Editorial and Data Presentation Modifications**

**Reviewer #1:** I recommend "Minor Revision" before accept the article to publishing.

*Answer*: Thank you for the comment.

**Reviewer #2:** (No Response)

**Reviewer #3:** Line 6: needs rephrasing – 2 diagnostic tests are recommended, not all are ELISAs and not all have high sensitivity and specificity (the authors also make these points in lines 95-97

Line 76 “individuals”

*Answer*: We accept the recommendation of the reviewer for more clarity relating to the protocol using the reference tests and the rapid diagnostic tests.

This is the same observation as that of Reviewer #2.

The following paragraph was replaced:

which recommends that the first test (total/raw antigen ELISA) used in the sample algorithm confirms a sensitivity ≥ 98%; because of this, in the present study, the ELISA Chagas III GrupoBios® test was used [29], and as a complementary test, according to the recommendations of INS, the recombinant antigen or synthetic peptide ELISA test was used with a specificity of ≥ 98%, after first using a Chagas ELISA IgG+IgM I Vircell® test [30,31]. In the case of conflicting results, as a third proof of principal or of different antigens than the first two tests, an IgG antibody technique for *T. cruzi* was used for IFI [32]: when the results of this technique were positive, the case was considered to be confirmed. All tests were completed using the serum following the manufacturer's instructions and the results were interpreted as either positive or negative.

By the paragraph:

All samples were analyzed using the ELISA Chagas III GrupoBios® tests [informed sensitivity, 100%; specificity, 100%] [29] and with the Chagas ELISA IgG+IgM I Vircell® tests [informed sensitivity, 100%; specificity, 98%] [30,31]. In cases in which discordant results were found between the two ELISA tests, an IIF test was done [32], which uses epimastigotes of the Colombian *T. cruzi* strains DTU and TcI as antigens, when the results of this technique were positive, case was considered to be confirmed. The tests were done in serum samples in the LDSP, following the manufacturer’s instructions (L 172 a 179 ).

*Answer*: The term “individuals” was revised.

-------------------------------------------------------------------------------------

**Summary and General Comments**

**Reviewer #1:** There are really different results in the literature regarding rapid diagnostic tests for the detection of anti-t.cruzi antibodies. As the authors themselves say, this can be the result of several factors. “These differing results could have been influenced by the environment in which the RDT's were done (e.g. laboratory or field tests), for the nature of the samples utilized in the evaluation (e.g. due to how the blood was stored), or due to the antigenic variation of the strains of the parasite in the different regions in which the CD was transmitted”

Furthermore, we consider that an important point must be taken into account: which quality control criteria were used as a reference in this study?. According to the mentioned logarithm, the reference was made to methodologies (two ELISAS and 01 IFI) but  not quality control criteria was adopted. Some works cited actually used strict CC criteria. For example, in reference #44, a blind sample panel was used by all laboratories that participated in the evaluation. The authors cite that 4 different analysts performed the tests. Does this laboratory participate in external quality assessment programs for anti-t.cruzi tests? Does this laboratory have an internal quality control, with samples of low reactivity, of use to validate daily runs?

It would be interesting if the authors comment  two aspects, regarding the positivity of the leishmania positive samples and the reinforcement of quality control procedures adopted.  Perhaps could add some information about them in the text. Although the work should be published.

The same observation was made in the Conclusions section.

*Answer*: We accept the recommendation of the reviewer and the following text has been added:

The tests were done in serum samples in the LDSP, following the manufacturer’s instructions; the laboratory complies with the internal quality control standards of the method, using material for quality control of the MQC and with external quality controls and aptitude tests done by the INS and Proasecal. The results of the tests were interpreted as either positive or negative (L 178 a L 182).

.

*Answer*: We accept the recommendation of the reviewer and the following text has been added:

In order to evaluate cross-reactivity with leishmaniasis, participants from the municipalities of Otanche and San Pablo de Borbour were convened, they were diagnosed by the program of diseases transmitted by vectors of the LDSP with cutaneous leishmaniasis confirmed through the identification of the parasites from smears from cutaneous lesions using microscopy (L 190 to L 194).

**Reviewer #2:** This is a relevant, correctly designed and implemented study that show consistent results. Further implementation research on the use of these RDTs is in my opinion warranted.

The findings of this study are of interest for other NTDs. Optimization of CD diagnostics is also of interest for clinical practice in general both in endemic and non-endemic countries.

The authors may want to consider some suggestions:

- Transmission of CD via transplantation is missing.

*Answer*: We accept the reviewer’s recommendation. Transmission of Chagas disease by transplant was included in the following paragraph:

and transmission from organ transplants due to the fact that, as with transfusions, transplants with organs from donors who live in endemic areas may contain the parasites and disseminate parasitosis to a immunosuppressed host [3] (L 66 to 68).

- Use “patient management” instead of “patient treatment” (not all CD patients need anti-parasitic treatment).

*Answer*: We accept the recommendation of the reviewer. The word “treatment” was replaced with “management” (L 244 a L 245).

- We know that RDTs do not need refrigeration but what about stability and shell life under field temperature and humidity conditions?

*Answer*: We accept the recommendation of the reviewer. The following paragraph was included:

Although the RDT’s can be stored at ambient temperatures between 8-30ºC during their useful lifespans, it is important to keep in mind that exposing them to temperatures greater than 30ºC may affect the performance of the tests. The cassette devices must be used immediately after their removal from the bag in order to minimize their exposure to humidity. [29,30]. (L 377 a L381).

- A word on the other diagnostic tests for CD such as Western blot, chemiluminescence and molecular techniques could be useful, since they may play a role if the RTDs become routine.

*Answer*: We accept the recommendation of the reviewer. We have included other tests for the diagnosis of CD.

Serological diagnosis in the chronic phase of CD is based on the detection of circulating antibodies through conventional reactions such as the enzyme-linked immunosorbent assay (ELISA) test, Indirect Immunofluorescence (IIF), Indirect Hemagglutination (IHA), Chemoluminescence (ChLIA) and Western blot/Immunoblot [18]. (L 102 to L 106).

- The authors may want to discuss how the two RDTS performance fit into the ASSURED criteria (see <https://www.who.int/bulletin/volumes/95/9/16-187468/en/>).

*Answer*: We accept the recommendation of the reviewer. The ASSURED criteria were consulted and are included in the discussion. We have added the reference where the criteria were consulted in reference #51.

Both RDT's evaluated in the present study complied with the ASSURED (Affordable, Sensitive, Specific, User-friendly, Rapid and robust, Equipment-free and Deliverable to end-users) criteria which are used as references for the identification of diagnostic tests that are most appropriate for areas with limited resources [51] (L 382 to L 385).

Reference 51. Kosack CS, Page AL, Klatser PR. A guide to aid the selection of diagnostic tests. Bull World Health Organ. 2017 Sep 1;95(9):639-645. doi: 10.2471/BLT.16.187468. Epub 2017 Jun 26. PMID: 28867844; PMCID: PMC5578377.

- I am not an English native speaker myself, therefore I would however recommend checking the validity of some terms with an English native speaker.

*Answer*: We accept the recommendation of the reviewer. Several terms have been changed to their corresponding acronyms in English.

A review was made of all the terms used in the article

IFI was replaced with IIF

IC was replaced with CI

**Reviewer #3:** It is not really clear why the study was done as these tests, a noted in the Discussion, have been evaluated in a number of other studies and with widely varying (and lower than the current study) sensitivities.  This is yet another result - to what end/better understanding?  The authors offer reasons why results in these different tests could differ, but make no conclusions that would help in REALLY knowing the utility of these tests.

*Answer*: We accept the recommendation of the reviewer. We have changed a paragraph in the discussion in order to broaden the information provided about the utility of RDT’s in the Colombian context. The response is included within the remainder of the discussion.

The findings of the present study show the excellent results that can be obtained using RDT's in terms of validity, safety, and reproducibility in the Colombian epidemiological context: they can be used in the field using samples of whole blood and they may be used in endemic areas in which laboratory installations necessary to realize conventional tests such as the ELISA, the IHA, or the IIF are unavailable; and in which infections with *T. cruzi* and leishmaniasis may be superimposed without presenting cross-reactions. The results obtained in the evaluation of precision diagnostics of the PDR’s provide data that can be used for decision making in terms of the utilization of these tests within the diagnostic algorithm used in Colombia [18] for the diagnosis of chronic CD, thus improving access to treatment as quickly as possible beginning at the primary health care level (L 399 to L 408).

-------------------------------------------------------------------------------------

The Word “blind” was added in order to provide more clarity with regard to the fact that the study analysts did not have any knowledge of the provenance of the tests.

A blind prospective observational study was developed (L 141)

-------------------------------------------------------------------------------------

**Figure Files:**

Answer: Figure 1 was loaded in the PACE tool.

**Date Requirements**

Answer: The data used for development of the article is included in the support materials.

**S1 Table.** Complimentary table of qualitative and quantitative results of rapid diagnostic tests and standard reference tests utilized in the study.

**Reproducibility**

Answer: The laboratory protocol was deposited in protocol.io

Identifier (DOI): http://[dx.doi.org/10.17504/protocols.io.bttpnnmn](https://dx.doi.org/10.17504/protocols.io.bttpnnmn)
